# A New Public Health Tool for Risk Assessment of Abnormal Glucose Levels

**Published:** 2010-02-15

**Authors:** Guozhong He, Tetine Sentell, Dean Schillinger

**Affiliations:** California Diabetes Program. Dr He is also affiliated with the University of California, San Francisco.; University of Hawaii at Manoa, Honolulu, Hawaii; University of California San Francisco, Center for Vulnerable Populations at San Francisco General Hospital, California Diabetes Program University of California, San Francisco, and California Department of Public Health, Sacramento, California

## Abstract

**Introduction:**

Self-reported prediabetes and diabetes rates underestimate true prevalence, but mass laboratory screening is generally impractical for risk assessment and surveillance. We developed the Abnormal Glucose Risk Assessment-6 (AGRA-6) tool to address this problem.

**Methods:**

Self-report data were obtained from the 1,887 adults (18 years or older) in the National Health and Nutrition Examination Survey (NHANES) 2005-2006 with fasting plasma glucose and oral glucose tolerance tests. We created AGRA-6 models by using logistic regression. Performance was validated with NHANES 2005-2006 data by using leave-1-out cross-validation. Standard performance characteristics (sensitivity, specificity, predictive values, area under receiver-operating characteristic curves) were assessed, as was the potential efficiency of the models to reduce laboratory testing in screening efforts.

**Results:**

Performance was good for all models under testing conditions. Use of the AGRA-6 in screening efforts could reduce laboratory testing by at least 30% when sensitivity is maximized and at least 52% when sensitivity and specificity are balanced.

**Conclusion:**

The AGRA-6 appears to be an effective, feasible tool that uses self-reported data compatible with the Behavioral Risk Factor Surveillance System to assess population-level prevalence, identify abnormal glucose levels, optimize screening efforts, and focus interventions to reduce the prevalence of abnormal glucose levels.

## Introduction

Hyperglycemic conditions are a major public health problem, affecting an estimated 40% or more of the US adult population (1). Rates of hyperglycemic conditions, however, are not evenly distributed across the US population; they vary by race, ethnicity, age, sex, and other social and place-based factors. Prevalence rates for communities with different demographic characteristics vary (2). Numerous health risks, such as cardiovascular disease, kidney failure, and vision loss, are associated with abnormal glucose levels (3). Substantial health risks are associated not only with levels high enough to be classified as diabetes but also with the intermediate zones of glucose intolerance, termed prediabetes (4-6). The health consequences of prediabetes and diabetes can be limited with exercise, diet, and medication (7), and such measures can prevent progression from prediabetes to diabetes (3). Cases of hyperglycemia should be identified so that interventions can be focused and effective health planning provided, yet cases of abnormal glucose are difficult to identify through self-report. Nearly 90% of people who have prediabetes and 40% of those who have diabetes (1,8) are not aware of their clinical condition. These people may be asymptomatic yet vulnerable to complications (1), and may be less likely to undertake prevention efforts than those with a diagnosis (8,9).

Although all levels of abnormal glucose have health implications, the severity and scope of clinical outcomes vary by specific subtypes (10). The health problems associated with overt diabetes, which affects almost 13% of the US population (1), include stroke, heart disease, kidney and eye diseases (3), and premature death. An estimated 30% of the US population have prediabetes; this population has a slightly higher risk for heart disease than do those who do not have prediabetes (4). They also have a significantly higher risk for developing diabetes (11,12). Prediabetes can be diagnosed from impaired fasting glucose (IFG) or impaired glucose tolerance (IGT), though these diagnoses carry somewhat different risks. Isolated IFG is associated with a slight increase in premature death compared with normal glucose tolerance, whereas IGT is not (3). However, IGT is more costly to treat than IFG (13) and carries a slightly higher risk for heart disease (4). People with both IFG and IGT appear to have the greatest risk of developing diabetes (12) and incur the greatest costs (13). Because of differences in clinical outcomes, some have suggested that distinct preventive recommendations should accompany the different types of prediabetes (4).

Predictive algorithms provide a means to estimate rates of abnormal glucose levels (particularly unrecognized abnormal glucose levels) in specific populations when laboratory data are not available, and they offer a method for determining individual risk that can be used to better focus screening efforts. A number of attempts have been made to quantify abnormal glucose risk by using such methods (3,14-20). These models have proved useful in both clinical practice and estimation of population illness (3,16,18) but have a  limitation: none provides a way to quantify the clinically relevant measures of abnormal glucose that may be important in health surveillance and intervention. Most models focus on diabetes risk specifically (14,16,18,20), often using samples atypical of the general US population or requiring knowledge of clinical or laboratory data (15,18-20). This makes them impractical for surveillance and risk assessment for most measures of abnormal glucose in most US populations. A few recent models include a measure of both undiagnosed diabetes and prediabetes (14,15,17,19), but these do not distinguish between the types of prediabetes (14,17,19), consider specific populations (15), or focus exclusively on quantifying individual prediabetes risk for ease of use in clinical settings (17).

The purpose of this study was to improve on previous research by using a nationally representative sample of US adults to create a predictive algorithm for 6 of the clinically relevant measures of abnormal glucose (IFG, IGT, prediabetes, IFG/IGT, undiagnosed diabetes, and total abnormal glucose) by using readily available self-report data. To maximize the usefulness of this instrument for public health work, we employed variables available from the Behavioral Risk Factor Surveillance System (BRFSS), administered yearly by US states and territories.

## Methods

### Data source

This study used the public dataset from the nationally representative National Health and Nutrition Examination Survey (NHANES) 2005-2006 (21), which oversampled minority populations. Households were randomly assigned to morning or evening examination. Morning examination included a fasting plasma glucose test (FPG) and an oral glucose tolerance test (OGTT); 1,887 participants aged 18 years or older had valid measures for both FPG and OGTT. More detailed methods of the NHANES 2005-2006 (21) and the laboratory tests can be found elsewhere (22).

### The Abnormal Glucose Risk Assessment-6 models

We developed 6 models to estimate all clinically relevant measures of abnormal glucose levels. Model 1 estimates IFG. Model 2 estimates IGT. Model 3 estimates prediabetes (either IFG or IGT). Model 4 estimates what we term "high-risk prediabetes" (both IFG and IGT). Model 5 estimates undiagnosed diabetes. Model 6 estimates total abnormal glucose, which includes prediabetes, undiagnosed diabetes, and diagnosed diabetes.

### Study samples

For the first 4 models — all estimates of prediabetes risk — we excluded the 308 people who met the criteria for frank diabetes, whether they were aware (201) or unaware (107) of this diagnosis. This left 1,579 people (of the 1,887) for estimates for models 1 through 4. For model 5, estimating undiagnosed diabetes, we excluded people who were aware they had diabetes (201), yielding a sample of 1,686 people. For the model estimating total abnormal glucose prevalence (model 6), we included all 1,887 adults with FPG and OGTT scores.

### Abnormal glucose variables

Operational definitions of IFG, IGT, prediabetes, and diabetes were developed on the basis of current diagnostic criteria of the American Diabetes Association (23). IFG was defined by an elevated FPG concentration (≥100 and <126 mg/dL). IGT was defined by an elevated 2-hour plasma glucose concentration (≥140 and <200 mg/dL) after a 75-g glucose load on the OGTT. Prediabetes was defined as having either IFG or IGT. High-risk prediabetes was defined as having both IFG and IGT; the definition was based on previous work about the increased risk of this situation (4,12,13). Diabetes was defined as having a fasting plasma glucose of 126 mg/dL or more or a 2-hour plasma glucose above 200 mg/dL. Total abnormal glucose was defined as having prediabetes of any form, undiagnosed diabetes, or diagnosed diabetes. Diagnosed diabetes was determined by individual self-report and did not include gestational diabetes, which was not assessed in the 2005-2006 NHANES.

### Predictor variables

On the basis of a literature review, we identified 11 self-reported predictor variables that were available in the NHANES and BRFSS and were known to be associated with diabetes risk for possible inclusion in each Abnormal Glucose Risk Assessment-6 (AGRA-6) model. Demographic variables included age (continuous 18-85 y), sex, self-reported race/ethnicity, and educational attainment. Behavioral variables included smoking status and participating in any leisure-time physical activities. Health condition variables included body mass index (BMI) (continuous and truncated from ≤10 to ≥100 to avoid outliers), history of hypertension, use of hypertension medication, high cholesterol, and family history of diabetes.

### Statistical analysis

From these possible variables, we derived optimal logistic prediction models by using the Akaike information criterion (AIC), which selects a model that maximizes predictive power while minimizing the number of predictive variables (24). For each of the 6 outcome variables, a unique, optimal predictive model was built from the set of potential predictive variables. The main statistical analyses were performed with SAS version 9.1 (SAS Institute, Inc, Cary, North Carolina). Multiple imputations were performed with SRCware version 1.0 (University of Michigan, Ann Arbor, Michigan). All of the models took into account the complex survey design.

### Model validation and performance

We validated the final models with the leave-1-out cross-validation (LOOCV) method. The LOOCV uses a single observation from the whole sample as the validation data, and the remaining observations as the training data. This process is repeated until each observation in the entire sample is used once as the validation data. The sensitivity, specificity, and positive and negative predictive values were obtained for all 6 models under LOOCV testing conditions. The area under receiver-operating characteristic curves (AUC) provides a single value that indicates the discrimination of the model (ie, its ability to identify true risk) at all possible values that could be chosen as cut points to distinguish risk from nonrisk.

In practical applications, however, specific cut points must be chosen to distinguish risk from nonrisk. Whether that cut point should prioritize identifying true positives, true negatives, or some balance between the 2 depends on the objective of the analysis and the budget of the program doing the analysis. For instance, when algorithms are used for screening purposes, it would generally be more desirable to find all cases of prediabetes, at the cost of some false positives. In this situation, the cut point delineating risk from nonrisk should be set to maximize sensitivity (finding all true positives) over specificity (identifying only true negatives). A positive finding of risk would then be followed by a laboratory test. For surveillance, on the other hand, the goal would typically be to strike a balance between types of error (false positives and false negatives). A higher specificity cut point is generally more cost-effective unless clinical priorities dominate (such as use of higher sensitivity cut points to find gestational diabetes).

Therefore, to maximize the usefulness of the AGRA-6, we present the predictive characteristics of each AGRA-6 model for 2 thresholds: 1) the high-sensitivity threshold, where a cut point is selected so that sensitivity is reached to about 0.9 (approximately 90% of positive cases will be correctly identified), and 2) the balanced-sensitivity/specificity threshold, where a cut point is selected so that sensitivity and specificity are equal. This will enable users of the tool to determine optimal cut points on the basis of local or programmatic needs and resources.

## Results

Descriptive statistics for this nationally representative sample of adults aged 18 or older are summarized for each of the possible predictor variables and for all 6 of the abnormal glucose outcome variables (Table 1).

The final AGRA-6 models (Table 2) show that the number of the 11 possible predictor variables differed by outcome variable. For instance, the optimal model for high-risk prediabetes included only 4 of the possible predictor variables, whereas the optimal model for total abnormal glucose included 7 variables. In no final model were both hypertension and use of hypertension medication included together.

We examined the performance and efficiency of each model at both the high sensitivity and balanced sensitivity and specificity cut points (Table 3). All models had AUC values within the acceptable range (0.72-0.80), and most were higher than 0.75. Under the high sensitivity threshold, the 2 models (IFG and prediabetes) that would deem the most people to be high risk would still predict 30% of the total population to be no-risk and would not require testing from them. The high sensitivity model for undiagnosed diabetes would predict only 34% of the population at high risk and would avert testing for 66% of the population. If model 6 was used as the first step in a 2-stage screening for total abnormal glucose prevalence in a population under this cut point, it would capture 90% of true positives while keeping 33% of the population from laboratory testing.

Under the balanced sensitivity and specificity threshold, all models had sensitivity values from 0.64 to 0.77 and specificity values from 0.67 to 0.73. If the AGRA-6 models were used with survey data such as BRFSS data to estimate abnormal glucose prevalence in a region, the AGRA would be able to accurately predict about 70% of various clinical classifications of both total abnormal glucose cases (sensitivity) and noncases (specificity) in that region. If model 6 was used to predict the total abnormal glucose prevalence in a population under this cut point, it would misclassify 27% of true negatives. Model 6 would keep 52% of the population from laboratory testing.

## Discussion

The AGRA-6 is the first risk assessment tool to estimate 6 clinically meaningful measures of abnormal glucose including 4 distinct categories of prediabetes, undiagnosed diabetes, and total abnormal glucose prevalence. It is designed to be used with readily available self-reported data, particularly BRFSS data. The AGRA-6 offers these advantages while maintaining comparable performance to existing measures that include fewer outcome variables and/or necessitate clinical or laboratory data.

The AGRA-6 should prove helpful in efforts to achieve at least 3 public health goals. First, it could be useful for surveillance. AGRA-6 estimates have their own uses and can be coupled with geographic data to highlight neighborhoods and other localities where the prevalence of abnormal glucose is disproportionate. Health planners and advocates can also use these models to compare, for the first time, the prevalence of different types of prediabetes in their communities and, thus, different types of clinical risk. Current prediabetes prevalence estimates based on the BRFSS self-report of being diagnosed with prediabetes may miss nearly 90% of prediabetes cases. Second, the AGRA-6 could be useful for screening. One key implication of our study is that readily available data from various community and public health settings could be used to enhance the efficiency of mass screening to enable focused screening for prediabetes and previously undiagnosed diabetes. All of the models would reduce the need for testing to find true positives. Finally, the AGRA-6 can be useful for individual risk assessment. In clinical practice, the models could be incorporated into electronic medical records to produce risk estimates for individual patients for all of the 6 abnormal glucose levels. For the general public, the AGRA-6 is being developed into an online tool that can provide individual risk assessment for all 6 levels of abnormal glucose (www.caldiabetes.org).

The AGRA-6 provides 4 key advantages over previous work in this area. First, it predicts 6 of the clinically meaningful levels of abnormal glucose, whereas previous work has included only some of these outcomes. Second, it uses basic self-report data to generate predictions on the basis of actual laboratory findings. It does not require laboratory work or additional clinical information. Third, it is directly compatible with the BRFSS, providing a link to existing surveillance efforts in many locations. Fourth, it is based on a representative sample of the entire US adult population.

### Limitations

The AGRA-6 has some limitations. Computing devices — either personal computers or personal digital assistants — will generally be required to calculate risk models. This should not present a barrier for most AGRA-6 applications, but when these devices are unavailable or impractical, other tools (14,17,18) may be preferable even if they do not allow for the measure of as many subtypes of abnormal glucose risk.

Second, the AGRA-6 models have been validated by using data from the sample on which they were created, which may result in more overestimations of model performance than would be observed if the algorithms were tested in other data sets. We did this because there are no other comparable nationally representative data sets that contain laboratory tests for both FPG and OGTT. To minimize the impact of this approach, we used the LOOCV method, which is a method often used for creating testing data sets from training data sets (25).

Third, although these models were generated from a nationally representative US sample, they may not be appropriate for all US subpopulations and geographic areas or for many international populations (26). The performance of the AGRA-6 models may also vary by demographic subgroups (younger vs older, heavier vs lighter, different racial/ethnic groups), and a consideration of this variation for the AGRA-6 models and for other commonly used predictive models is an area for further study. Some of the predictive variables rely on prior access to care, including having a diagnosis of hypertension or high cholesterol and taking hypertension medication. Actual prevalence in people who lack access to care may thus be underestimated. Also, the available sample was not large enough to allow us to include Asians/Pacific Islanders as distinct subpopulations, leaving open a question regarding its usefulness for classifying risk in these groups.

Fourth, the AGRA-6 shares the limitations of any predictive model in that some people will be misclassified. Whether people are misclassified as false positives or false negatives can be manipulated to some degree by the chosen threshold levels used to delineate risk. In all public health surveillance and screening, there will be tradeoffs between precision and cost, and no option is infallible. The problems associated with misclassification must be weighed against the specific goals and budget of the program.

### Implications for public health practice

The health risks of type 2 diabetes can be mitigated through individual, community-based, and even structural and policy interventions (3). Lifestyle interventions can also prevent or delay the onset of type 2 diabetes among high-risk people, such as those with prediabetes (6). One major task for public health agencies and programs is to identify groups and individuals who would benefit from these interventions. The AGRA-6 allows public health organizations to identify populations and individuals who would probably benefit from these interventions and to facilitate cost-effective screening of these populations. This could further facilitate the allocation of public health resources for focused interventions to reduce the illness and death risks of prediabetes and diabetes in the United States. The AGRA-6 models should also prove useful for county, state, and national surveillance efforts to assess the progression of this epidemic.

## Figures and Tables

**Table 1:** Characteristics of Adults Aged 18 or Older Who Completed Both Valid Fasting Plasma Glucose and Oral Glucose Tolerance Tests, NHANES, 2005-2006 (n = 1,887)

**Demographic Characteristics**	**Mean (95% CI)**	**Unweighted n**
**Age, y **	45.7 (44.9-46.6)	1,887
**BMI (kg/m^2^)**	28.3 (28.0-28.7)	1,815^a^

	**% (95 % CI)**	**Unweighted n^b^**

**Female**	52.0 (49.2-54.8)	892
**Race/ethnicity**		
Non-Hispanic white	71.2 (69.0-73.4)	904
Non-Hispanic black	11.4 (10.2-12.6)	452
Hispanic	12.0 (10.6-13.4)	456
Other	5.4 (4.1-6.7)	75
**Education**		
Less than high school graduate	16.6 (14.8-18.5)	445
High school graduate	26.1 (23.5-28.6)	423
Some college	57.3 (54.5-60.1)	819
**Smoking status**		
Current	24.5 (21.9-27.0)	362
Former	25.4 (22.9-27.9)	458
Never	50.1 (47.3-53.0)	866
**No leisure-time physical activity**	23.7 (21.4-26.1)	496
**Health conditions**		
Told at risk for diabetes	15.2 (13.0-17.3)	240
Medical history of CVD	10.7 (9.0-12.3)	223
Hypertension	30.3 (27.8-32.8)	621
Take medication for hypertension	24.5 (22.2-26.8)	518
High cholesterol	41.2 (37.9-44.5)	518
Diabetes in family	42.1 (39.2-45.0)	734
**Abnormal glucose status**		
Impaired fasting glucose (IFG)	23.4 (21.1-25.7)	446
Impaired glucose tolerance (IGT)	13.4 (11.6-15.3)	262
Prediabetes (IFG **or** IGT)	29.0 (26.5-31.5)	558
High risk for prediabetes (IFG **and** IGT)	7.8 (6.4-9.3)	150
Undiagnosed diabetes	5.0 (3.8-6.1)	106
Total abnormal glucose^c^	41.4 (38.7-44.2)	866

Abbreviations: NHANES, National Health and Nutrition Examination Survey; CI, confidence interval; BMI, body mass index; CVD, cardiovascular disease.

a Subjects were removed as outliers if BMI was ≤10 or ≥100.

b Totals vary due to missing data.

c Includes prediabetes, undiagnosed diabetes, and diagnosed diabetes.

**Table 2:** AGRA-6 Models^a^ for Predicting Abnormal Glucose Levels by Self-Reported Data

**Model**	**Predictive Equation^b^**
**Model 1: Impaired fasting glucose (IFG)**	Log (odds of IFG) = -4.1389 + (0.0366 × [age]) + (0.0419 × [BMI]) + (1.0038 × [male]) –(0.5430 × [NH white]) + (0.0373 × [NH black]) – (0.5875 × [MX American]) + (0.5737 × [HTN meds])
**Mode 2: Impaired glucose tolerance (IGT)**	Log (odds of IGT) = -4.5598 + (0.0422 × [age]) + (0.0269 × [BMI]) + (0.4958 × [hypertension]) – (0.3946 × [physical activity]) + (0.3449 × [high cholesterol])
**Model 3: Prediabetes (PDM)**	Log (odds of PDM) = -4.1268 + (0.0444 × [age]) + (0.0408 × [BMI]) + (0.8448 × [male]) – (0.4041 × [NH White]) + (0.1888 × [NH Black]) – (0.4438 × [MX American]) + (0.3685 × [hypertension])
**Model 4: High-risk prediabetes (HRP)**	Log (odds of HRP) = -6.4083 + (0.0461 × [age]) + (0.0394 × [BMI]) + (0.8947 × [hypertension]) + (0.5428 × [high cholesterol])
**Model 5: Undiagnosed diabetes (UDM)**	Log (odds of UDM) = -7.6961 + (0.0543 × [age]) + (0.0382 × [BMI]) + (0.9804 × [hypertension]) + (0.2723 × [less HS grad]) + (0.8583 × [HS grad])
**Model 6: Total abnormal glucose (TAG)**	Log (odds of TAG) = -4.4298 + (0.0486 × [age]) + (0.0493 × [BMI]) + (0.7158 × [male]) – (0.4303 × [NH white]) + (0.1420 × [NH black]) – (0.4397 × [MX American]) + (0.7064 × [hypertension]) + (0.1981 × [high cholesterol]) + (0.4113 × [less HS grad]) + (0.0910 × [HS grad])

Abbreviations: AGRA, Abnormal Glucose Risk Assessment; BMI, body mass index; NH, non-Hispanic; MX, Mexican; HTN meds, hypertension medications; HS grad, high school graduate.

a The coefficients in the equations are derived from the optimal logistic prediction models using the Akaike information criterion.

b The log (odds of event) is defined as log [*P*/(1-*P*)], where *P* is the probability of an event. Substitute categorical terms with 1 if yes and 0 if otherwise.

**Table 3:** Performance of AGRA-6 Predictive Models at 2 Possible Thresholds: High Sensitivity^a^ and Balanced Sensitivity and Specificity^b^

Conditions	Model 1, IFG	Model 2, IGT	Model 3, PDM	Model 4, HRP	Model 5, UDM	Model 6, TAG
**Area under the ROC curve**	0.72	0.78	0.75	0.78	0.80	0.80
**High sensitivity threshold**						
Cut point	0.15	0.10	0.20	0.05	0.05	0.25
Sensitivity	0.89	0.85	0.89	0.90	0.77	0.90
Specificity	0.40	0.53	0.40	0.56	0.69	0.47
Positive predictive value	0.36	0.26	0.45	0.18	0.15	0.59
Negative predictive value	0.90	0.95	0.87	0.98	0.98	0.85
Proportion who would screen at high risk^c^	70	54	70	49	34	67
Proportion of laboratory tests avoided	30	46	30	51	66	33
**Balanced sensitivity and specificity threshold**						
Cut point	0.30	0.15	0.35	0.10	0.05	0.40
Sensitivity	0.64	0.74	0.69	0.71	0.77	0.73
Specificity	0.68	0.68	0.67	0.73	0.70	0.73
Positive predictive value	0.44	0.32	0.53	0.21	0.15	0.69
Negative predictive value	0.83	0.93	0.80	0.96	0.98	0.76
Proportion who would screen at high risk	41	39	46	31	34	48
Proportion of laboratory tests avoided	59	61	54	69	66	52

Abbreviations: IFG, impaired fasting glucose; IGT, impaired glucose tolerance; PDM, prediabetes; HRP, high-risk prediabetes; UDM, undiagnosed diabetes; TAG, total abnormal glucose; ROC, receiver-operating characteristic.

a Finding true positives is prioritized.

b Finding true positives is balanced with finding true negatives.

c Identifies the percentage of those tested who would have a model-predicted risk score that is greater than or equal to the cut point. In a screening situation, this would be the percentage of people who would be recommended for further testing.
